# Associations between dietary habits and body mass index with gut microbiota composition and fecal water genotoxicity: an observational study in African American and Caucasian American volunteers

**DOI:** 10.1186/1475-2891-8-49

**Published:** 2009-10-21

**Authors:** Volker Mai, Quintece M McCrary, Rashmi Sinha, Michael Glei

**Affiliations:** 1 Microbiology and Cell Science, Emerging Pathogens Institute, University of Florida, Gainesville, FL, USA; 2 Food Science and Technology, University of Maryland Eastern Shore, Princess Anne, MD, USA; 3 DCEG, National Cancer Institute, Bethesda, MD, USA; 4 Institute for Nutrition, Department for Nutritional Toxicology, Friedrich-Schiller-University, Jena, Germany

## Abstract

**Background:**

African Americans (AA) suffer from an increased incidence and mortality of colorectal cancer (CRC). Environmental exposures including dietary habits likely contribute to a high burden of CRC, however, data on the dietary habits of AA is sparse. Diet might change the composition and the activities of the intestinal microbiota, in turn affecting fecal genotoxicity/mutagenicity that is thought to be associated with carcinogenesis.

**Methods:**

We assessed dietary habits by food frequency questionnaire and by food records in 52 AA and 46 CA residents of the Eastern Shore of MD. Fecal microbiota composition was determined using 16S rRNA based methods and fecal genotoxicity measured using the Comet assay.

**Results:**

AA reported an increased intake of heterocyclic amines and a decreased dietary intake of vitamins including vitamin D (p < 0.05) that correlated with differences in fecal microbiota composition but not fecal genotoxicity. Intake of dietary fiber, calcium, total fat and heterocyclic amines correlated with differences in microbiota composition. Total bacterial counts/g of stool and raw counts of Bacteroides were increased in AA. In contrast to a previous study, BMI was not associated with proportions of Bacteroides.

**Conclusion:**

Dietary habits of African Americans, including increased HCA intake and decreased vitamin D intake might at least partially contribute to CRC through modifications of gut microbiota composition that result in changes of the intestinal milieu.

## Introduction

Colorectal cancer (CRC) is thought to be strongly associated with environmental exposures including diet. Components ingested through the diet are a major source for exposure of the colonic epithelium to mutagenic compounds that may cause both initiation of cell transformation and tumor progression [1,2]. The objective of the current study was to determine if dietary habits, previously shown to differ between African Americans (AA) and Caucasian Americans (CA), effect the composition of gut microbiota and result in differences in fecal water cytotoxicity/genotoxicity.

AA suffer from an increased incidence and mortality of CRC in comparison to CA (American Cancer Society: Statistics for 2008, ). Although the underlying causes for these differences are not well established it is plausible that differences in dietary habits in AA affect the colonic environment by increasing exposure to mutagens directly as well as indirectly through changes in the composition of the metabolically active gut microbiota [3]. In a previous large study of associations between diet and CRC in AA and CA in North Carolina differences between the two racial groups were detected in the intake of micro- as well as macronutrients [4-6]. This study also suggested that associations between diet and CRC might differ between the two racial groups. One observation in the above study was an increased intake of heterocyclic amines (HCAs) by AA. HCAs such as 2-amino-1-methyl-6-phenyl-imidazo[4,5-b]pyridine (PhIP), and 2-amino-3,8-dimethylimidazo[4,5-f]quinoxaline (MeIQx), and also benzo[a]pyrene (Bap) a polycyclic aromatic hydrocarbon (PAH), are possible humancarcinogens formed during meat cooking. Large amounts of HCA are formed with longer cooking times, internal temperatures of between 150°C and 200°C, and greater external charring [7], typically achieved with cooking methods such as barbeque.

Humans harbor in their guts a complex intestinal microbiota that varies in its composition between individuals. Although recent studies have shed some light on the complexity of the gut microbiota in a limited number of individuals, the extent of microbiota diversity and associations with dietary habits are still poorly understood [8]. Tremendous advances in our understanding of the composition and activities of the gut microbiota have recently been made, however, we still do not fully understand the degree of complexity or the dynamics of the human gut microbiota [9,10]. It is now well established that the human gut microbiota is phylogenetically as well as metabolically very diverse and makes important contributions to the physiology of its human host [11,12]. Eloquent studies have shown communication between gut microbiota and the human host, a requirement of gut microbiota for appropriate priming and development of the immune system and associations between gut microbiota and obesity mediated by the induction/suppression of various human factors [13-17]. The host microbiota can have profound effects on nutrient acquisition and sequestering, immune priming and reactivity, as well as direct effects on carbohydrate and other compound levels in the systemic circulation [18,19]. The microbiota has long been suspected to be associated with health as well as with diseases including inflammatory bowel diseases and CRC, recent data supports an association between reduced microbiota diversity and Crohn's disease [20].

Fecal water, the water-soluble fraction obtained as the supernatant after high speed centrifugation of total feces, reflects the luminal content of both risk factors and protective factors [21]. Cytotoxicity/genotoxicity of fecal water is a useful biomarker in studying the impact of environmental factors on exposure of the gut to carcinogens and the modification of this exposure by dietary habits [22-24]. Cytotoxicity/genotoxicity of fecal water can be assessed *in vitro *by exposing cultured human colon cells, followed by assessment of cell viability and DNA damage in single cells by the Comet assay [25]. Fecal water genotoxicity has previously been shown to be related to colon carcinogenesis in animals [26]. In human studies, fecal water genotoxicity was higher after a diet rich in meat as compared to a diet enriched in fiber [27]. Supplementation with probiotic yoghurts has been shown to reduce fecal water genotoxicity [28]. There also appears to be an effect modification by smoking status as supplementation with bread enriched with prebiotics and antioxidants reduced the fecal water genotoxicity in non-smokers but not in smokers, an effect which differed by the status of GST M-polymorphisms [29].

In this study we investigated if differences in dietary habits between the two groups are associated with cytotoxicity/genotoxicity of fecal water and with fecal microbiota composition.

## Materials and methods

### Study design

#### Target population

We included subjects that were at least 40 years of age, self identified as being of AA or CA descent and able to provide informed consent and information regarding dietary history as well as basic demographic data. We excluded subjects that had a prior diagnosis of cancer (other than skin cancer); suffered from inflammatory bowel disease; had chronic diarrhea or acute diarrhea within the past 4 weeks; were hospitalized or used systemic antibiotics within the past 4 weeks. A total of 98 subjects, 52 AA and 46 CA were enrolled. To evaluate associations between obesity and microbiota composition all 14 lean (BMI <25) and 14 randomly chosen obese subjects (BMI >30) were selected. The study was approved by the Institutional Review Boards at UMB and UMES.

#### Study logistics

Subjects were approached about the study through local community groups. After enrollment, subjects received a study kit that included all questionnaires as well as a stool collection kit. After filling out the questionnaires subjects kept a four day food record and collected the first stool sample after its completion.

#### Assessment of diet and medical history

Subjects completed the self-administered Block 98 food frequency questionnaire (FFQ) that assessed their dietary habits [30], and a specific questionnaire assessing consumption of meat and its preparation (MMQ) [31]. Subjects also kept a food record of all foods and drinks consumed for four consecutive days. Medical history and demographic information was assessed using a scan able questionnaire specifically developed for this study.

#### Stool collection

After completing a four day food record subjects collected a freshly voided stool sample. The stool sample was transported to the laboratory in a plastic bag containing an ice pack within six hours. Upon arrival in the laboratory each sample was immediately homogenized and fixed for FISH analysis or frozen -80°C for all other analyses.

#### Analytical cohort

From the 98 subjects that completed the study protocol we removed subjects with low quality dietary data, those that skipped more than 10 questions on the FFQ and those that reported a daily calorie intake of less than 400 or more than 5200 calories. 42 AA and 40 CA remained in the FFQ/MMQ analysis and 39 AA and 38 AA in the Food Record analysis. All 14 lean study subjects (BMI <25) and 14 randomly chosen obese subjects (BMI >30) were included in the studies of the association between microbiota and obesity.

### Microbiota analysis

#### DNA Extraction

DNA was isolated from 200 mg of stool for each sample by using a modified QIAmp DNA Stool Mini kit (Qiagen, Cat. No. 51504, Valencia, CA). Noted modifications were the addition of 5 glass beads to the initial buffer solution to allow better homogenization of the stool while vortexing and the addition of 0.3 g zirconia beads (Biospec Products Inc., Cat. No. 11079101z, Bartlesville, OK) followed by 3 min of shaking in a Mini-BeadBeater (Glen Mills Inc., Clifton, NJ), prior to the addition of the Inhibit-EX tablet, to enhance the lysis of bacterial cell walls. Purified DNA samples were eluted in a final volume of 50 μl and stored at -70°C until analyzed.

#### Denaturing Gradient Gel Electrophoresis (DGGE)

A 457-bp fragment from the V6 to V8 region of the bacterial 16S rDNA gene was amplified with primers U968-GC (5' CGC CCG GGG CGC GCC CCG GGC GGG GCG GGG GCA CGG GGG GAA CGC GAA GAA CCT TAC) and L1401 (5' GCG TGT GTA CAA GAC CC), as described by Zoetendal *et al*. [32]. The GC clamp facilitates separation by DGGE. DGGE was performed in an 8% (wt/vol) polyacrylamide gel with a denaturing gradient ranging from 40% to 50% at the top and bottom of the gel, respectively (100% denaturing conditions were defined as 7 M urea and 40% formamide). After electrophoresis (16 h, 65 V, 60°C), the gels were stained with SYBER Green (Novex, San Diego, CA) and scanned/analyzed with Quantity One and Diversity Database software (Bio-Rad, Hercules, CA).

#### Fluorescent in situ hybridization (FISH)

Aliquots (0.5 ml) of homogenized feces were added to PBS (4.5 ml), and the samples were prepared for FISH analysis as described previously [33]. Briefly, the mixtures were processed by vortexing them with 3-5 glass beads for 2 min, removing fecal debris by centrifugation at low speed, fixing the bacteria-containing supernatant fluids overnight in PBS containing 3% paraformaldehyde, and storing aliquots of the fixed bacterial preparations at -70°C until hybridization was performed. Hybridization was performed by applying aliquots (10 μl) of appropriate dilutions of the fixed bacterial preparations to gelatin-coated microscopic slides, fixing the specimens to the slides with 95% ethanol, and hybridizing with 10 ng of the appropriate probe/μl, using the conditions described previously [33-35]. The following probes were used: (i) Bac303 for *Bacteroides *and *Prevotella *[36], (ii) Erec482 for eubacteria, clostridia and ruminococci belonging to *Clostridium *cluster XIVa [33], (iii) Bif164 for bifidobacteria [37], (iv) LAB158 for lactic acid bacteria [38]. After briefly rinsing with distilled water, slides were air-dried rapidly with compressed air and mounted with Vectashield containing DAPI (4',6-diamidino-2-phenylindole) (Vector Labs, Cat. No. H-1200, Burlingame, CA). DAPI and Cy3/FITC positive cells were enumerated in a total of 12 fields at two different dilutions per sample at 100× magnification using a Zeiss axioscope-40 epifluorescence-equipped microscope (Zeiss, Jena, Germany). The proportions of the two bacterial groups were calculated by dividing the number positive for the specific probe by the total bacteria as determined by DAPI. DAPI was used as it allows for the enumeration of total bacteria without the need for a separate hybridization.

#### Quantitative PCR (qPCR)

qPCR analysis was performed on a subset of 14 lean (BMI<25) and 16 obese subjects (BMI>30) in duplicate using a qPCR Core kit (Eurogentec, Cat. No. RT-SN10-05NR, San Diego, CA) on a Stratagene MX3000P (La Jolla, CA) in 12.5 μl reaction volumes consisting of 2 μl DNA template (diluted 1:80 in water), 1 × Reaction Buffer, 200 μM dNTP mix, 30 pM forward and reverse primers, 0.025 U/μl HotGoldStar Taq Polymerase, 1 × SYBR Green dye, 30 nM ROX passive reference dye (Stratagene, Cat. No. 600546, La Jolla, CA). The following primers and conditions were used: 1) all eubacteria (V3 F: 5'-CCTACGGGAGGCAGCAG-3';R: 5'-ATTACCGCGGCTGCTGG-3', 56°C, 3 mM MgCl_2_); 2) Bacteroides-Prevotella-Porphyromonas group (Bac F: 5'-GGTGTCGGCTTAAGTGCCAT-3'; R: 5'-CGGA(C/T)GTAAGGGCCGTGC-3', 68°C, 3 mM MgCl_2_); Clostridium coccoides-Eubacterium rectale group (Erec F: 5'-CGGTACCTGACTAAGAAGC-3'; R: 5'-AGTTT(C/T)ATTCTTGCGAACG-3', 55°C, 4 mM MgCl_2_) [39]. qPCR conditions were as follows: 10 min at 95°C followed by 40 cycles of 95°C for 30 s, annealing for 1 min (see primer specific temperature above), and extension at 72°C for 30 s.

qPCR standard curves covering the range observed in the samples were generated using a lab internal DNA standard derived from a human stool sample. The proportions of the two groups were calculated by dividing the number positive for the specific primer set by the total number of bacteria determined using the universal V3 primer set.

### Fecal water analysis

Fecal samples were homogenized using a glass rod. For preparation of the fecal water (FW), the feces were centrifuged (18500 rpm for 2 h at 4°C) and the supernatants were collected. After a further centrifugation step (13000 rpm for 10 min at 4°C) to obtain a clear solution, the liquid phases were quantified, the pH-values were determined with a pH meter, and the samples were aliquoted and stored at -80°C until use.

#### Human tumor cell line HT29

The human colon carcinoma cell line HT29, obtained from the American Tissue Culture Collection (Rockville, MD, USA), was used to test toxicity of the FWs [40]. Cells were kept frozen in liquid Nitrogen until thawed and grown at 37°C in a (95%) humidified incubator (5% CO_2_) in Dulbecco's Modified Eagle Medium (DMEM, Gibco BRL, Eggenstein, Germany) supplemented with 10% fetal calf serum, penicillin (50 U/mL) and streptomycin (50 μg/mL). Passages 22 to 29 (cytotoxicity and genotoxicity studies) and 40-50 (challenge assay) were used for the experiments.

#### Cytotoxicity assay

To determine the effective dose of FW the cell suspensions were initially incubated with 5%, 10% or 20% of FW. All following assays were performed using 20% concentration to reach a measurable DNA damage level. FW was added to 100 μl cell suspension containing 4 × 10^5 ^HT29 cells. The suspensions were incubated for 30 min in a shaking water bath at 37°C and cytotoxicity was determined using the trypan blue exclusion assay.

#### Genotoxicity assay

DNA damage was measured in 1 × 10^5 ^cells suspended in low melting point agarose on microscope slides with the single cell microgel electrophoresis assay, also known as the "Comet assay" [41] as previously described [42]. 50 images/slide were evaluated by measuring the percentage of fluorescence in the tail.

#### Challenging treatment to evaluate the antigenotoxic potential

The Comet assay was also used to determine if a pretreatment with fecal water leads to a modified genotoxic effect of H_2_O_2_. Cells were pretreated with 20% fecal water (30 min, 37°C) and subsequently incubated with 75 μM H_2_O_2 _for 5 min at 4°C. Viabilities were determined with the trypan blue exclusion test and the remaining cells were analyzed with the Comet assay as described above.

### Statistics

Multivariate regression analysis (SAS) was used to explore associations between multiple exposure factors and microbiota composition. Shannon Wiener index was used to calculate microbiota diversity of the DGGE profiles. Unpaired t-test and one-way ANOVA (Microsoft Excel version 2003) were used to calculate means and variation and for establishing two-sided significance levels (p < 0.05).

## Results

### Dietary analysis

After removal of subjects with low quality data 82 subjects, 42 AA and 40 CA, were retained in the analytical cohort for the FFQ based analysis of differences in dietary habits between the two racial groups. Both groups were similar in age and gender distribution. AA had a higher mean weight and body mass index but lower income and lower level of education (Table 1). AA consumed a diet lower in the percent of calories from fat and higher in percent of calories from carbohydrates (Table 2). AA also had a lower intake of supplemental vitamins including vitamin D. Although total intake of HCA did not differ between the groups, the intake of MeIQx and PhIP, two HCA formed during meat preparation at high temperature, was increased in AA (p < 0.05).

**Table 1:** Study demographics in African American (AA) and Caucasian American (CA) participants

	**AA**	**CA**
#	52	46

Mean age (years)	51.2	52.3

Sex (male)	27%	35%

Mean weight	199lb	177lb

Mean BMI	32	28

Income <30,000US	48%	33%

College degree	33%	61%

BMI is body mass index (weight/height^2 ^in kg/m^2^).

**Table 2:** Differences in diet between AAs and CAs as determined by FFQ

	**AA (42)**	**CA (40)**	**p-value**
Total calories (kcal)	1918	1861	0.8

Protein (g)	65	69	0.6

Fat (g)	77	82	0.6

Carbohydrates (g)	248	214	0.19

% kcal from protein	13.8	15.1	0.09

% kcal from fat	35.2	39.2	**0.01**

% kcal from carbs	52.4	46.2	**<0.01**

Dietary fiber (g)	17.6	16.7	0.7

Fruit (servings)	1.5	1.5	0.9

Vegetable(servings)	3.1	3.8	0.24

Dairy (servings)	0.68	1.43	**<0.01**

Calcium (mg)	608	774	0.07

*Supplements*			

Calcium (mg)	108	418	**<0.01**

Magnesium (mg)	19	42	**0.02**

Vitamin A (IU)	1200	2700	**0.03**

Vitamin C (mg)	140	380	**<0.01**

Vitamin D (IU)	77	166	**0.02**

Vitamin E (a-TE)	46	157	**<0.01**

*Heterocyclic amines*			

Total (ng/day)	190	128	0.1

MelQx (ng/day)	47	25	**0.04**

PhIP (ng/day)	126	78	0.07

Dimelqx (ng/day	2.8	2.1	0.2

Bap (ng/day)	14	22	0.1

PhIP is 2-amino-1-methyl-6-phenyl-imidazo[4,5-b]pyridine, MeIQx is 2-amino-3,8-dimethylimidazo[4,5-f]quinoxaline, Dimelqx is 2-amino-3,4,8-trimethylimidazo[4,5-f]quinoxaline Bap is benzo[a]pyrene.

Data from the four day food records mostly supported the findings from the FFQ analysis, albeit fewer associations reached statistical significance (data not shown). Specifically, the differences in percent calories from fat and carbohydrates as well as the decreased intake of vitamin D in AA were confirmed with this secondary tool.

### Fecal water analysis

FW analysis was performed in a subset of 21 AA and 22 CA subjects for which we had a sufficient amount of fecal sample. We successfully extracted an average of 18.6% FW from the fecal samples. No difference in yield was observed between the samples from AA and CA. The mean pH of the FW was 6.9, the observed difference between AA (6.7) and CA (7.1) was borderline significant (p = 0.05) when both genders were combined and significant in females alone (AA: 6.7, CA: 7.2; p < 0.05). To determine the appropriate dose of fecal water for the genotoxicity assay, the cell suspensions were incubated with 5%, 10% or 20% FW. FW induced DNA damage in a dose dependent manner (p = 0.052). However, even at the highest dose of 20% FW, which was used in all subsequent studies, we observed only a weak increase in measurable DNA breaks.

**Figure 1:**
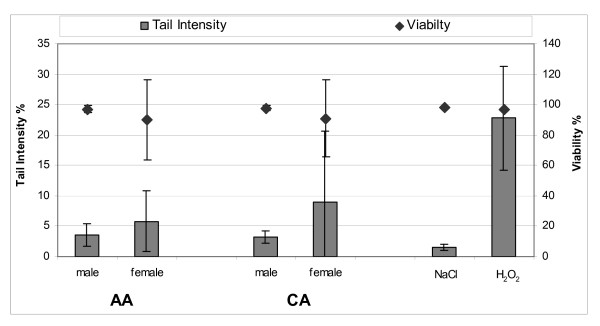
**DNA damage and viability of HT 29 cells after incubation with FW**. AAs (n = 21), CAs (n = 22), males and females. NaCl -- negative control; H_2_O_2 _-- positive control.

We observed no differences in fecal water induced cytotoxicity or genotoxicity between the two racial groups as both, the tail intensity and the viability of the HT 29 cells showed no significant differences between the two groups (Figure 1). When we analyzed the proportion of undamaged cells with a tail intensity ≤6% as an alternative measure of genotoxicity we detected only a small and statistically insignificant difference between the two racial groups that suggested a trend towards slightly reduced genotoxicity in fecal waters from AA. Furthermore the results showed a significantly increased genotoxicity in FW from females that was not associated with cell viability (Figure 1). BMI, age and diet including fat intake and dietary levels of heterocyclic amines were not associated with either genotoxicity or viability (data not shown). The large variation associated with the genotoxicity assay would not have allowed us to detect any small or medium effects with the limited number of subjects included in this study. To evaluate potential protective effects of FW we analyzed the effects of preincubation of HT29 cells with fecal water before challenge with H_2_O_2 _and measurement of its genotoxicity, using the remaining fecal waters from 33 subjects. Although H_2_O_2 _clearly induced DNA breaks preincubation of the HT29 cells with fecal waters did not affect the degree of DNA damage (data not shown).

### Microbiota analysis

**Figure 2:**
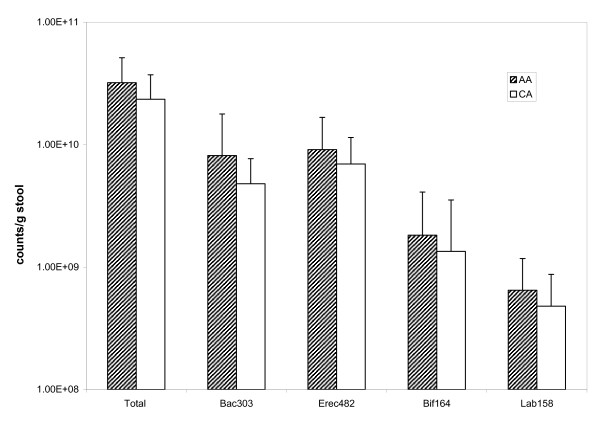
**Comparison of microbiota between AA and CA**. Fixed stool samples were hybridized with probes directed against Bacteroidetes (Bac 303), Clostridium Cluster XIVa (Erec482), Bifidobacteria (Bif164) and Lactic Acid bacteria (Lab158) and enumerated by FISH as described in Materials and Methods. Total bacterial counts were determined using DAPI.

We first analyzed overall microbiota composition by qualitative DGGE profiling, an efficient but crude method for assessing microbiota diversity (a measure of species richness and their distribution). We did not detect a difference in overall diversity with any of our measured variables (data not shown). Based on DGGE profiles, the mean Shannon-Wiener diversity index in all samples, a measure of overall microbiota diversity, was 2.3 with a SD of 0.2 and a range between 2.0 and 2.7. In contrast, quantitative microbiota analysis by FISH revealed some differences between the two racial groups. The total number of bacteria/g of stool was higher in AA when compared to CA (Figure 2). These differences, although statistically significant on the decimal scale, were not significant after log transformation. Although total number of Bacteroidetes that hybridized to our probe were increased in AA (Figure 2) the proportions of Bacteroidetes or Clostridia cluster XIVa (Firmicutes) that were targeted with the respective probes did not differ between AA and CA. We detected suggestive associations between dietary intake and quantities of bacterial groups. Subjects that consumed high amounts of calories from fat harbored fewer Clostridia. Consumption of dietary fiber was positively associated with numbers of LAB, which are thought to be beneficial to health. Grain fiber and fiber from fruits and veggies but not fiber from beans appeared associated with LAB (data not shown). Subjects with higher intake of HCAs had higher amounts of Clostridia cluster XIVa. All of these potential associations between exposure of interest and microbiota reached statistical significance (p < 0.05) only in the initial analyses of raw data, however, after log transformation of bacterial counts to achieve a more normal distribution none of the associations remained significant.

**Figure 3:**
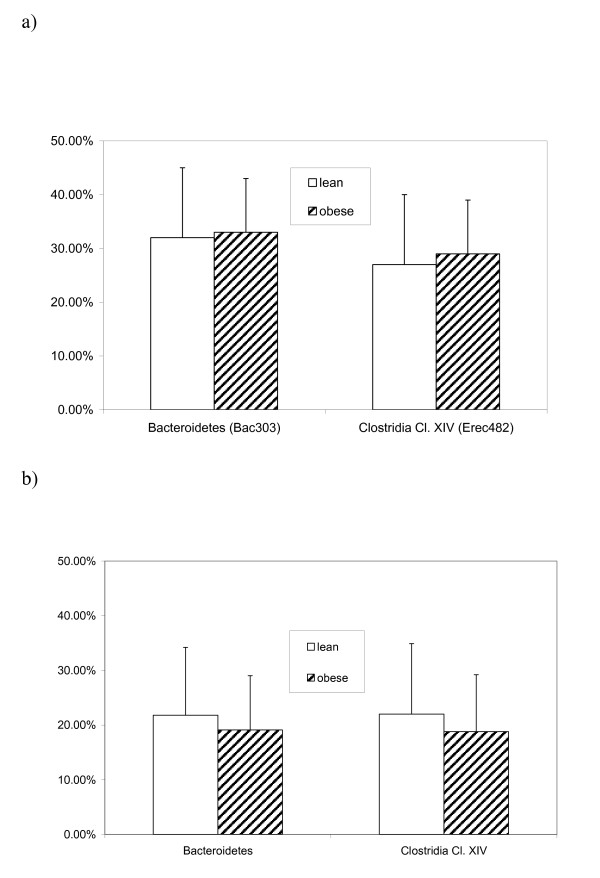
**Proportions of bacteroidetes and Clostridia Cluster XIVa in obese vs. lean subjects as determined by a) FISH and b) qPCR analysis**.

When we analyzed the proportions of Bacteroidetes and Clostridia cluster XIVa (Firmicutes) in all 14 lean study subjects (BMI<25) compared to 14 matched obese subjects (BMI>30), we failed to detect an association with BMI by either FISH (Figure 3a). The apparent lack of associations between obesity and the proportions of Bacteroidetes and Clostridia cluster XIVa in our study population was confirmed using a second quantitative approach, qPCR (Figure 3b).

## Discussion

Our analysis of differences in dietary habits between AA and CA is consistent with previous reports that suggested an increased intake of HCAs and a decreased intake of micronutrients in AA [4-6]. Although our study was limited in size, the agreement of two independent dietary assessment methods (FFQ and food record) increases confidence in our observation. As both HCA intake and vitamin D levels have been associated with carcinogenesis [7,43] there appears to be an opportunity for dietary prevention to reduce the burden of cancer in AA.

Exposure of HT29 cells to FW from subjects in this study did induce DNA damage. However, although the two groups differed in various exposures, including BMI and dietary habits, we did not detect significant differences in responses to FW from the two groups. Our study was limited in size and measurement of cytotoxicity and genotoxicity included a large degree of variation. The observation that FW from females appear to exhibit higher genotoxicity is not consistent with the hypothesis that FW genotoxicity is associated with CRC risk, as US females have lower CRC rates than males. Although we used twice as much fecal water (20%) compared to earlier studies [27,29], we observed only a weak increase in measurable DNA breaks. Future studies of associations between environmental factors and FW genotoxicity should be designed in larger human cohorts preferably with controlled exposures to reduce variation.

The observation that total bacterial counts/g of stool and numbers of Bacteroides counts were increased in AA is suggestive rather than confirmatory. Soft large stools of rural Africans have been considered as representing low colorectal cancer risk in early observations of the effects of dietary fiber intake [44]. A higher water content of stools would reduce bacterial densities and thus harder stools might contain larger densities of bacteria. We collected only one spot stool sample from each subject and variation in microbiota composition over time appears significant [45,46]. The same qualifications need to be made when interpreting our findings that intake of fiber, HCA and calories from fat affected microbiota composition. Although we have shown earlier that dietary interventions in mice as well as in humans can affect microbiota composition [45,47], similar data from observational studies is sparse [3]. There has been significant recent interest in potential associations between gut microbiota and obesity, specifically a decrease proportion of bacteria belonging to the Bacteroidetes among obese [15,48-50]. Our data does not support that hypothesis as AA in our study had a higher BMI that correlated with higher, not lower, numbers of Bacteroidetes. A detailed quantitative study in a subset of lean and obese subjects from this study also failed to detect such an association. We used a different methodology (FISH and qPCR) compared to the at best semi-quantitative 16S rRNA based analysis frequently used by other groups. Our probes and primers admittedly do not cover Bacteroidetes species perfectly and due to differences in methodology results can't be compared directly to 16S rRNA based studies. However, recent work from other groups also appears inconsistent with a proposed association between lower proportions of Bacteroidetes and obesity [17,51]. The ongoing Human Microbiota Roadmap Project is aimed at improving our understanding of normal microbiota composition and its potential associations with health and disease . Our observations are consistent with the hypothesis that diet mediated differences in gut microbiota contribute to the observed increased risk of colorectal cancer in AA. Because diseases such as CRC can change the intestinal environment with likely impacts on microbiota composition, future studies with prospectively collected stool samples will be required to link microbiota composition with disease risk.

## Competing interests

The authors declare that they have no competing interests.

## Authors' contributions

VM designed the study, helped with study logistics, analyzed the data and wrote the manuscript. QM enrolled subjects, collected samples and performed the microbiota analyses. RS helped with the design of the dietary assessment and contributed to the HCA analysis. MG helped wit the study design, supervised the mutagenicity studies and wrote respective eparts of the manuscript. All authors read and approved the final manuscript.
